# Rapidly Worsening Infective Endocarditis With Severe Mitral Stenosis

**DOI:** 10.1016/j.jaccas.2024.102764

**Published:** 2024-11-20

**Authors:** Daiki Yoshiyama, Hiroyuki Morokuma, Kiyotaka Nagashima, Kouhei Baba, Masahide Shichijo, Nagi Hayashi, Kouki Jinnouchi, Manabu Itoh, Junji Yunoki, Keiji Kamohara

**Affiliations:** Department of Thoracic and Cardiovascular Surgery, Faculty of Medicine, Saga University, Saga, Japan

**Keywords:** infective endocarditis, mitral valve, transthoracic echocardiography, valve obstruction

## Abstract

Infective endocarditis (IE) rarely results in mitral stenosis (MS), but MS in patients with IE can be life-threatening. We present a case of prosthetic MS secondary to IE. A 69-year-old Japanese man underwent mitral valve replacement with a bioprosthetic valve 2 years previously. The patient presented with a 1-month history of illness, and we diagnosed prosthetic valve IE with severe MS and planned for time-sensitive surgery. However, the patient developed cardiogenic shock in response to prosthetic mitral valve obstruction while awaiting surgery. The patient then had to undergo emergency surgery. There are no management guidelines for IE-induced valve stenosis, whose treatment differs from that of valve regurgitation. Our literature review reveals that achieving survival in patients with MS secondary to IE is difficult without surgical intervention. Patients with MS caused by IE may require surgery, and specific criteria should be outlined in future guidelines.

## Visual Summary

**Figure:**
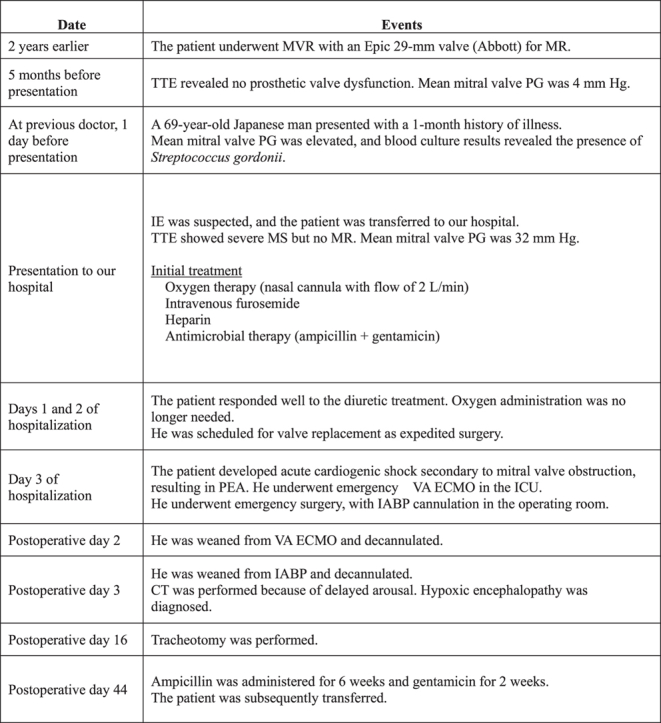

Infective endocarditis (IE) resulting in isolated valve stenosis is rare, and valve regurgitation is much more commonly observed. There are no guidelines regarding the treatment strategies for valve stenosis secondary to IE.^1^^,^^2^ The indication and timing of surgical intervention are usually left to the attending team in each case.

**Take-Home Messages**

• There is no guideline for valve stenosis following IE, and its management differs from that of valve regurgitation.
• Urgent surgery would be better to consider in patients with severe valve stenosis secondary to IE because the patient’s condition can abruptly deteriorate in response to valve obstruction.

Here we present a case of prosthetic mitral stenosis (MS) resulting from IE and rapidly progressive heart failure caused by prosthetic mitral valve obstruction while the patient was awaiting surgery.

## Case Summary

**Figure 1:**
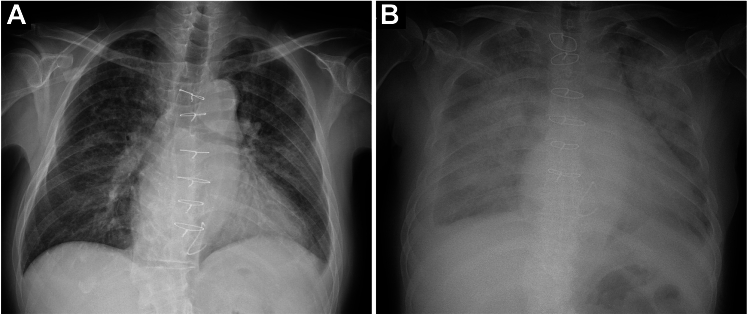
Chest Radiographs (A) At the initial visit, marked pulmonary congestion is visible. (B) After rapid decline, flash pulmonary edema is noted.

**Figure 2:**
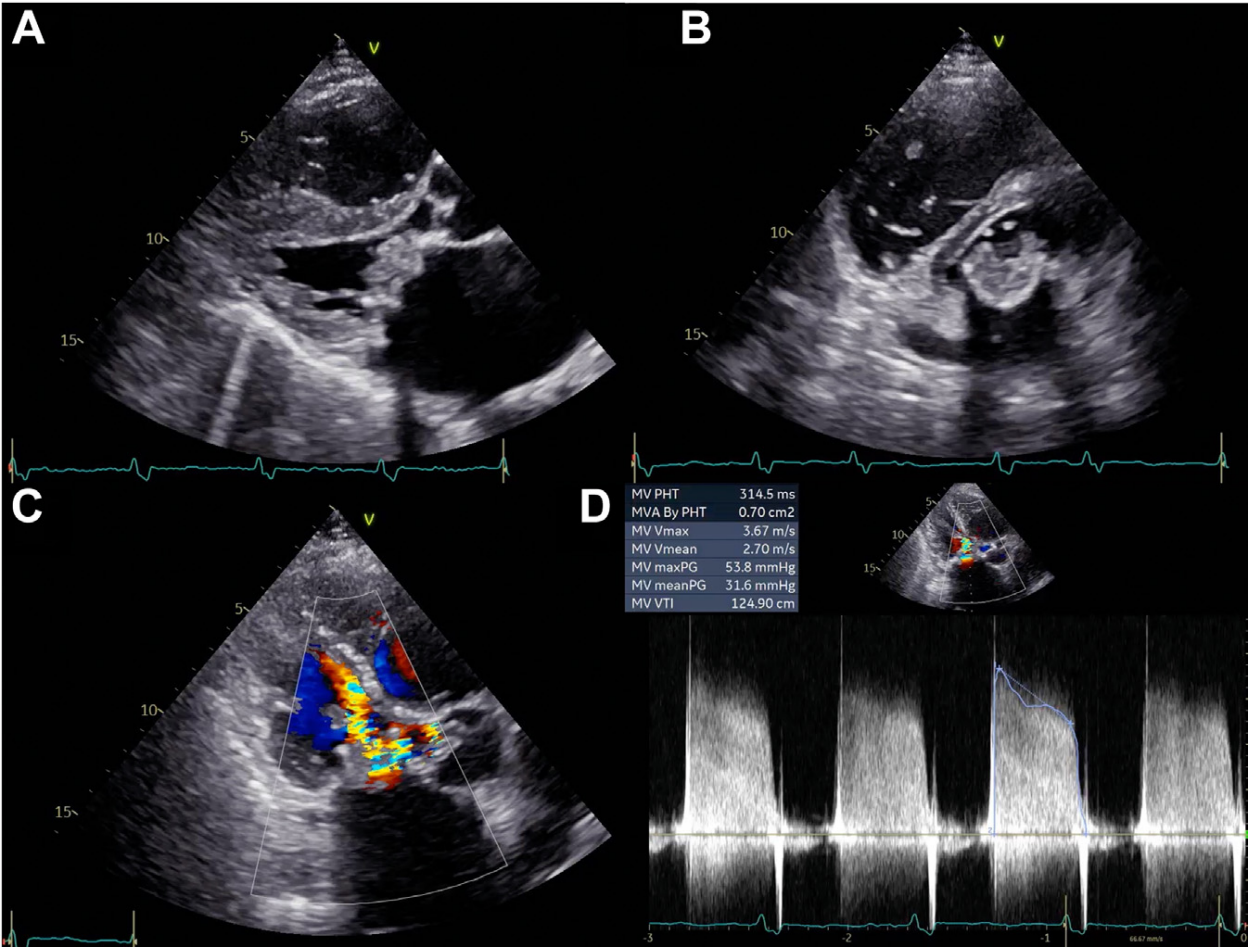
Transthoracic Echocardiographic Images (A and B) Transthoracic echocardiography reveals thickening of the mitral valve (MV) leaflets, (C) no mitral regurgitation at the color Doppler signal, and (D) increased transmitral flow (Video 1). MVA = mitral valve area; PHT = pressure half-time; Vmax = maximum velocity; Vmean = mean velocity; VTI = velocity-time integral.

A 69-year-old Japanese man presented to the clinic with a 1-month history of IE-related illness. He had undergone mitral bioprosthetic valve replacement with an Epic 29-mm valve (Abbott) for mitral regurgitation 2 years earlier. Five months earlier, transthoracic echocardiography (TTE) revealed no prosthetic valve dysfunction, and the mean pressure gradient (PG) was 4 mm Hg. Blood culture results detected the presence of *Streptococcus gordonii,* and the patient was transferred to our hospital (Saga University, Japan) with a diagnosis of prosthetic valve IE. The patient presented with a blood pressure of 94/62 mm Hg, and his pulse rate was 84 beats/min, his respiratory rate was 16 breaths/min, his temperature was 36.9 °C, and his oxygen saturation (Spo_2_) was 98% on a nasal cannula with a flow of 2 L/min. Physical examination revealed no murmurs or wet lung sounds. There were no embolic or petechial hemorrhagic findings suggestive of IE. A chest radiograph revealed pulmonary congestion (Figure 1A). Electrocardiography showed atrial fibrillation. Blood test results revealed elevated N-terminal pro–B-type natriuretic peptide levels at 56,434 pg/mL(normal: <125 pg/mL), a white blood cell count of 19,800/μL (normal: <9,700/μL), and a C-reactive protein level of 3.36 mg/dL (normal: <0.30 mg/dL). TTE revealed mitral valve leaflet thickening and reduced mobility, with a mean mitral valve PG of 32 mm Hg (Figures 2A to 2D, Video 1). The entire prosthetic mitral valve was thickened, with a maximum short diameter of 12 mm. Part of the vegetation was mobile. The patient was administered heparin and empirical antimicrobial therapy (ampicillin sodium and gentamicin).

**Figure 3:**
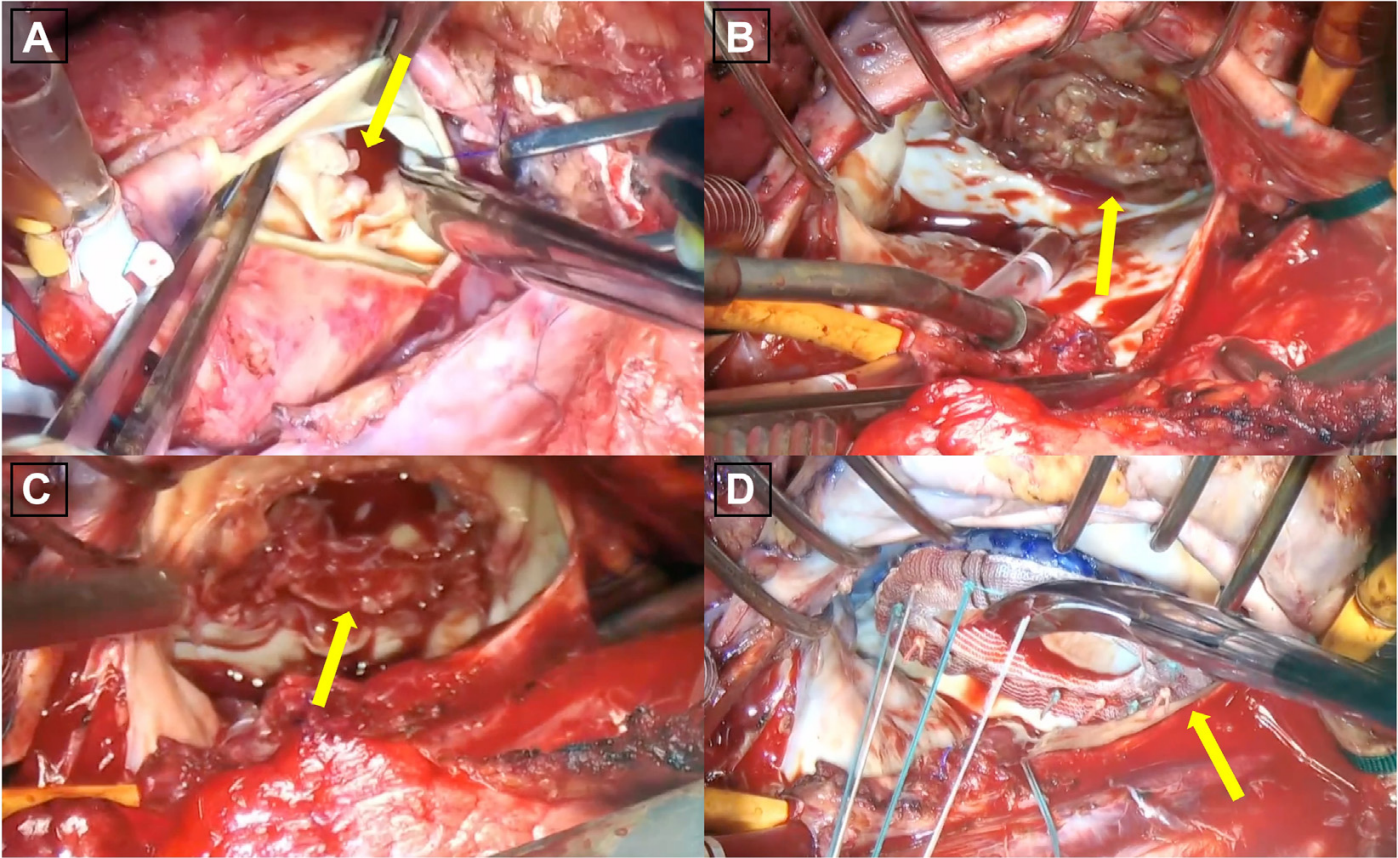
Intraoperative Images (A) Aortic valve vegetation (arrow). (B) Mitral valve vegetation (arrow). After (C) resection of the mitral valve (arrow), (D) mitral valve implantation (arrow) (Video 2).

The patient responded well to diuretic treatment. He was scheduled for valve replacement on the fourth day. On day 3 of his hospitalization, the patient’s condition abruptly deteriorated, with the development of severe hypotension and the onset of pulmonary edema (Figure 1B). Venoarterial-extracorporeal membrane oxygenation (VA ECMO) was initiated, and he underwent emergency surgery. During the operation, a vegetation was found on the aortic valve, with a small periaortic abscess. A massive vegetation covered the entire bioprosthetic mitral valve (Figures 3A to 3D, Video 2). The annulus was preserved. The patient underwent aortic and mitral valve replacements with Inspiris Resilia 23-mm and Mitris Resilia 29-mm prosthetic valves, respectively (Edwards Lifesciences). Pathologic examination of the mitral valve vegetation showed scant necrotic fibrous tissue in the background of abundant fibrin with acute inflammation, consistent with IE. An intra-aortic balloon pump (IABP) was inserted in the operating room.

The patient’s VA ECMO support was withdrawn within 2 days, and IABP support was withdrawn within 3 days. The patient’s postoperative course was complicated by a disturbance of consciousness, and we made a diagnosis of hypoxic-ischemic encephalopathy. Gentamicin was prescribed for 2 weeks and ampicillin for 6 weeks postoperatively. Postoperative TTE revealed good hemodynamics and no infection. The patient was subsequently transferred from our hospital.

## Why Beyond the Guidelines

IE-induced valve stenosis is not included in the Duke classification,^1^^,^^2^ and there are no treatment intervention guidelines.^3^^,^^4^ Therefore, it may take several days to diagnose the disease, and a treatment plan must be considered for each case. The heart failure and infection were well controlled with management, despite the severe MS, until a sudden worsening necessitated urgent (within 24 hours) surgery. IE-induced valve stenosis requires treatment guidelines different from those for valve regurgitation.

## Case Outcome and Follow-Up

Currently, the patient has no cardiac disability but still has neurologic dysfunction.

## Discussion

This case highlights the following aspects regarding IE-induced MS. First, acute deterioration may occur even in patients with controlled infection or heart failure. Second, apart from the severity of infection, the severity of damage to the stenotic valves should be considered.

IE-induced MS is rare, and a review of the literature from 1967 to the present in PubMed revealed 25 papers, 35 case reports of MS or acute and subacute obstruction of the mitral valve secondary to extensive vegetation, and 3 cases of diffuse thickening of the prosthetic valve leaflet reported previously.^3^, ^4^, ^5^, ^6^, ^7^ Of the 22 patients who underwent surgical intervention, 4 died (mortality rate, 18%). Furthermore, 12 of the 13 patients who did not undergo surgical intervention died (92%), and the status of the remaining patient was unknown because the clinical course was not reported. Achieving survival in patients with IE-induced MS is difficult without surgical intervention.

Among the 35 reported cases, at least 18 occurred in patients whose condition worsened quickly, with severe hypotension or shock. We summarized the 10 cases where the mean PG values of the mitral valve were measured (Table 1).^3^, ^4^, ^5^, ^6^, ^7^, ^8^, ^9^, ^10^ Patients with cases 1 to 3 had minor elevations in mean PG, but during medical management, the mean PG increased to >10 mm Hg, requiring intervention within a few days. For patients with cases 4 to 10, with the exception of 2 inoperable cases, all patients with a mean PG >15 mm Hg suddenly worsened and required emergency surgery. Patients with a mean PG >15 mm Hg required urgent surgery because of the high likelihood of sudden worsening while waiting for surgery. Even if the mean PG is low, there is a tendency for it to increase with conservative treatment. Thus, preparation for surgery should begin when the mean PG elevation is mild.

**Table 1:** Previously Reported Cases of Mitral Stenosis or Obstruction in Infective Endocarditis in the Literature

Case #	First Author	Year	Age, y	Sex	Previous Heart Disease	Bacteria	Mean PG of Mitral Valve, mm Hg	Size of Vegetation, cm	Site of Vegetation	Treatment	Surgical Indication	Days From TTE to Surgery	Outcome
1	Citrin^8^	1997	72	Male	PCI	Viridans *Streptococcus*	3→10	3.0 × 1.5	Anterior MV	Medical→expedited surgery	Increase in mean PG	5	Survived
2	Charney^5^	1993	58	Female	No	*Streptococcus faecalis*	Mild→12	2.0 × 3.0 flat vegetation	Posterior MV	Medical→expedited surgery	Increase in mean PG	Not stated	Survived
3	Roberts^9^	2019	63	Male	PCI	*Staphylococcus aureus*	8→14	Not stated	Not stated	Medical→expedited surgery	Increase in mean PG	2	Survived
4	Kobulnik^7^	2008	48	Female	No	*Staphylococcus aureus*	16	Not stated	Not stated	Medical→emergency surgery	Sudden decline secondary to valve obstruction	8	Survived
5	Tiong^6^	2002	51	Male	Post MVP for myxomatous disease	*Enterococcus faecalis*	18	Not stated	Not stated	Medical→emergency surgery	Inability to manage the hemodynamics medically	Not stated	Survived
6	Fernandes^10^	2021	28	Female	No	*Staphylococcus aureus*	18	Not stated	Not stated	Difficult to operate	—	—	Unknown
7	Bando^3^	2024	78	Male	Post AVR and MVR (Magna 21 mm, Edwards Lifesciences, + Epic 29 mm, Abbott)	*Cutibacterium acne*	18.4	Not stated	Not stated	Medical→emergency surgery	Heart failure uncontrolled	3	Survived
8	Hart^4^	2017	23	Female	Post MVR (Epic 25 mm, Abbott)	*Staphylococcus aureus*	22	Not stated	Not stated	Difficult to operate	—	—	Died after a few days
9	Charney^5^	1993	49	Female	No	*Streptococcus sanguis*	24	1.2 × 1.8	Posterior MV	Medical→emergency surgery	Sudden decline secondary to valve obstruction	Not stated	Survived
10	Yoshiyama	2024	69	Male	Post MVR (Epic 31 mm, Abbott)	*Streptococcus gordonii*	32	1.2	All valve cusp	Medical→emergency surgery	Sudden decline secondary to valve obstruction	4	Survived

AVR = aortic valve replacement; MV = mitral valve; MVP = mitral valve plasty; MVR = mitral valve replacement; PCI = percutaneous coronary intervention; PG = pressure gradient; TTE = transthoracic echocardiography.

## Conclusions

Treatment guidelines for IE-induced MS have not been described and should differ from guidelines for valve regurgitation. Patients with MS secondary to IE may not survive without surgical intervention. With the risk of sudden deterioration in response to valve obstruction, urgent surgery should be considered for patients with severe valve stenosis. Patients with MS caused by IE may require surgery for which specific criteria should be outlined in future guidelines.

**Table:** VISUAL SUMMARY. Timeline of the Case

Date	Events
2 years earlier	The patient underwent MVR with an Epic 29-mm valve (Abbott) for MR.
5 months before presentation	TTE revealed no prosthetic valve dysfunction. Mean mitral valve PG was 4 mm Hg.
At previous doctor, 1 day before presentation	A 69-year-old Japanese man presented with a 1-month history of illness. Mean mitral valve PG was elevated, and blood culture results revealed the presence of *Streptococcus gordonii*.
Presentation to our hospital	IE was suspected, and the patient was transferred to our hospital. TTE showed severe MS but no MR. Mean mitral valve PG was 32 mm Hg. Initial treatment Oxygen therapy (nasal cannula with flow of 2 L/min) Intravenous furosemide Heparin Antimicrobial therapy (ampicillin + gentamicin)
Days 1 and 2 of hospitalization	The patient responded well to the diuretic treatment. Oxygen administration was no longer needed. He was scheduled for valve replacement as expedited surgery.
Day 3 of hospitalization	The patient developed acute cardiogenic shock secondary to mitral valve obstruction, resulting in PEA. He underwent emergency VA ECMO in the ICU. He underwent emergency surgery, with IABP cannulation in the operating room.
Postoperative day 2	He was weaned from VA ECMO and decannulated.
Postoperative day 3	He was weaned from IABP and decannulated. CT was performed because of delayed arousal. Hypoxic encephalopathy was diagnosed.
Postoperative day 16	Tracheotomy was performed.
Postoperative day 44	Ampicillin was administered for 6 weeks and gentamicin for 2 weeks. The patient was subsequently transferred.

CT = computed tomography; ECMO = extracorporeal membrane oxygenation; IABP = intra-aortic balloon pump; IE = infectious endocarditis; ICU = intensive care unit; MR = mitral regurgitation; MS = mitral stenosis; MVR = mitral valve replacement; PEA = pulseless electrical activity; PG = pressure gradient; TTE = transthoracic echocardiography; VA = venoarterial.

## Funding Support and Author Disclosures

The authors have reported that they have no relationships relevant to the contents of this paper to disclose.

## Appendix

Video 1 Preoperative transthoracic echocardiography

Video 2 Surgical Video
